# Reduction in ARGs and Mobile Genetic Elements Using 2-Bromoethane Sulfonate in an MFC-Powered Fenton System

**DOI:** 10.3390/molecules30173502

**Published:** 2025-08-26

**Authors:** Weiye Wang, Jian Wei, Zhuang Guo, Xiaodong Bai, Yonghui Song

**Affiliations:** 1 State Key Laboratory of Environmental Criteria and Risk Assessment, Chinese Research Academy of Environmental Sciences, Beijing 100012, China; 13792735601@163.com (W.W.);; 2 Institute of Water Ecology and Environment, Chinese Research Academy of Environmental Sciences, Beijing 100012, China

**Keywords:** MFC-Fenton system, 2-bromoethane sulfonate, antibiotic resistance gene, MGEs, excess sludge

## Abstract

The integration of an MFC-powered Fenton (MFC-Fenton) system into the traditional anaerobic composting process can promote excess dewatered sludge (ES) decomposition. However, the antibiotic resistance gene (ARG) profiles in ES treated by MFC-Fenton systems remain poorly understood; in addition, the effect of adding 2-bromoethane sulfonate (BES, a methane inhibitor) during ES treatment using an MFC-Fenton system on ARG levels is largely unexplored. The present work focused on investigating the effects of BES and bioelectrochemical processes on ARG and MGE abundances and unraveling the ARG attenuation mechanism. According to our findings, adding BES promoted ARG reduction in ES in an MFC-Fenton system. The average ARG levels in the MFC-Fenton samples containing high BES contents (0.4 or 0.5 g BES/g VSS) markedly declined relative to those in samples containing lower BES levels. Moreover, macrolide transporter ATP-binding protein, macrolide-efflux protein, and *mac*B levels markedly decreased as BES levels increased. BES supplementation and bioelectrochemical assistance were crucial for altering the ARG composition in the MFC-Fenton system. Changes in the microbial community composition had the greatest effect on the variation in ARG composition. Furthermore, the *Actinobacteria* and *Firmicutes* levels accounted for 52.8% of the overall ARG variation. Among MGEs, plasmids, insertion sequences, and integrons showed lower levels within the sludge metagenomes. Typically, *sulI*, *sulII*, *tet*G, and *bla* TEM levels were positively correlated with metal resistance genes (MRGs), and their levels markedly declined following the MFC-Fenton process. Thus, the collective evidence indicates that BES synergizes with bioelectrogenesis to reduce ARG abundance.

## 1. Introduction

Antibiotic resistance genes (ARGs) have garnered significant global attention because they pose severe threats to environmental and public health [1]. Due to limited intestinal absorption in humans and animals, many ARGs are excreted and enter the environment [2]. Consequently, excess sludge contains the highest levels of ARGs and was deemed to be a key ARG reservoir [3]. Excess sludge possesses a high density and diversity of microbes, which can enhance ARG transfer via mobile genetic elements (MGEs, i.e., transposons), and its diffusion into environments accessible to humans has raised public health concerns [4,5].

ES contains many ARGs, including numerous human and livestock wastewater-derived ARGs, and antibiotic-resistant bacteria (ARB) [6]. WWTP-produced excess sludge can be dewatered to reduce the volume, giving rise to excess dewatered sludge (ES). Typically, the most common ES disposal approach is land application to recycle useful components. The ES disposal rates through land application are about 60% in the US and >50% in European Union countries such as the United Kingdom, France, and Belgium; however, the proportion of ES utilized in resource recycling in China is less than 40% [7,8].

ES is pretreated with anaerobic composting (AnC) prior to land application. This ES compost can then be used to produce soil conditioner or fertilizer to improve soil properties without consuming massive amounts of energy, and can stabilize soil organics [9,10]. However, during ES land application, ARGs are introduced into and disseminated within the soil. Thus, ARG levels in ES must be reduced prior to land application [3].

Traditional sludge composting cannot efficiently regulate ARG and MGE growth and dissemination due to the elevated sludge bacterial community abundance and diversity [11,12]. Xia et al. found that the elevated ARG levels and diversity in ES confer resistance to most antibiotic classes, making ES land application to soil undesirable [13]. Integrating a bioelectrochemical process into conventional AnC accelerates the process through bioelectrogenesis [14]. More specifically, the sludge degradation rate is increased since simple organic compounds are utilized by the exoelectrogens in the bioelectrochemically assisted AnC system [15]. Wang et al., Su et al., and Rago et al. revealed that integrating a microbial fuel cell (MFC)-powered Fenton (MFC-Fenton) system with composting produced electricity and adding a methane inhibitor such as 2-bromoethane sulfonate into the substrate enhanced the power and current densities [16,17,18]. Zhao et al. found that an MFC-Fenton system can decrease the carbon/nitrogen ratio and salinity and could generate more power [19]. Based on our prior results [20,21], compared to traditional AnC, the application of an MFC-Fenton system to treat ES could promote compost maturation and electric energy generation; moreover, the elevated cathode surface area and proton exchange area could increase the power density and promote organic matter decomposition.

An MFC-Fenton system is advantageous in minimizing ARG proliferation and dissemination. It can markedly change resistome and microbiome structures while reducing the overall, functional, and phylogenetic ARG diversity, as well as network complexity in biofilms, thereby reducing the ecological risk related to ARGs and multidrug-resistant bacterial evolution [22]. Compared with traditional anaerobic treatments, long-term acclimation can markedly decrease the total ARG and MGE copy numbers within an MFC-Fenton system anode’s biofilms [12]. Relative to long-term low-concentration cefuroxime treatment within traditional sequencing batch biofilm reactors, an MFC-Fenton system can decrease the number of copies of *OXA*-1, *OXA*-2, and *OXA*-10, three typical β-lactam ARGs [3]. In contrast, in research on pure ARB model bacteria, electron and current shuttles negatively affected ARG and ARB dissemination within an MFC-Fenton system. Based on the above findings, the application of an MFC-Fenton system to reduce ARBs and ARGs is associated with a certain risk, and it is crucial to improve the bioelectrochemically assisted AnC system for reducing ARGs within ES compost. However, the ARG and MGE profiles and levels in ES treated using MFC-Fenton systems remain largely unexplored.

Introducing additives (such as copper, biochar, surfactants, superabsorbent polymers, red mud, natural zeolite, silver nanoparticles, coal gasification slag, zero-valent iron, manure, and superphosphate) into sludge composting can enhance ARG removal [23,24]. ES composting carried out with natural zeolite (1% wet weight), a metal additive, for 183 days reduced the total number of ARG copies by 1.5% due to conjugation reduction and heavy metal co-selection due to the natural zeolite, with the number of *bla*_CTX-_M, *bla*_TEM_, *erm*B, *ere*A, and *tet*W copies decreasing by 0.3–2 logs [3]. One study analyzed how zero-valent iron (Fe^0^) affects the reduction in multiple typical tetracycline ARGs and the integrase gene *intI*1 in thermophilic anaerobic co-digestion of kitchen waste and ES. At 5 g/L Fe^0^, there were net reductions of 1.44–3.94 log units for multiple typical tetracycline ARGs [5]. In terms of using agricultural waste as an additive, a 39-day chicken manure composting experiment was conducted, which included four tests using 7 t of raw materials (wet weight) containing 210 kg of ferrous sulfate and 350 kg of natural zeolite; ARGs were reduced by 72.2% and 86.5% [11]. The mean reduction rates in poultry manure and chip and 5% agricultural crop biochar, and poultry manure and chip were 0.61 and 1.49 log units [3]. At 30 days following the addition of bamboo carbon and bamboo vinegar acid to the AnC process, the total MGE and ARG copy numbers markedly declined by 94.75–98.40% and 76.58–81.49%, respectively [25]. Manure-demolition waste co-composting was used as an approach to make manure safe to use as a soil amendment, which was found to decreased the *tet*M and *tet*W contents over time (*p* < 0.05) [26].

As a 2-mercaptoethane sulfonic acid (coenzyme M, CoM) analogue, BES can only be produced by methanogens, and it is required for the final step in the CH_4_ metabolism pathway [18]. CoM is initially transformed into methyl-CoM (CH_3_-S-CoM), which interacts with coenzyme B (CoB) after catalysis by CH_3_-S-CoM reductase to generate CH_4_ (Equation (1)). BES can persistently suppress methanogenesis in anaerobic environments, thus efficiently enhancing anode electricigen enrichment and domestication. In MFC-Fenton systems where excess sludge is used as the substrate, Wang et al. [20,27] demonstrated that adding BES to the anodic chamber in the MFC-Fenton system dramatically suppressed methane generation and elevated the coulombic efficiency (CE). Moreover, the addition of BES and the use of alkaline environments also faciliated extracellular electron transfer, enabling organic matter decomposition and physically destroying extracellular DNA in BES-treated ES. However, there are no studies on whether BES can play a role in reducing ARGs in ES through bioelectrochemistry.

CH_3_-S-CoM + CoB → CoM-S-S-CoB + CH_4_↑ (1)

Therefore, in this study, a dual-chamber MFC-Fenton system was used to investigate how adding BES affects ARG reduction in bioelectrochemically assisted AnC of ES. Specifically, the present work focused on (1) investigating how BES and bioelectrochemical processes affect ARG diversity and abundance; (2) analyzing how BES and bioelectrochemical processes influence MGE abundance; and (3) unraveling the ARG reduction mechanism through examining the contributions made by MRGs, the microbial community, and MGEs. The results suggest that BES combined with an MFC-Fenton system can be applied to reduce ARGs in ES. This study investigated the synergistic effect of BES and a microbial fuel cell-assisted Fenton system on the attenuation of ARGs and MGEs in excess dewatered sludge, with the aim of elucidating the dominant mechanisms driving ARG reduction, including microbial community shifts and metal resistance gene interactions.

## 2. Results and Discussion

### 2.1. ARG Levels and Diversity

#### 2.1.1. ARG Types, Levels, and Diversity

**Figure 1:**
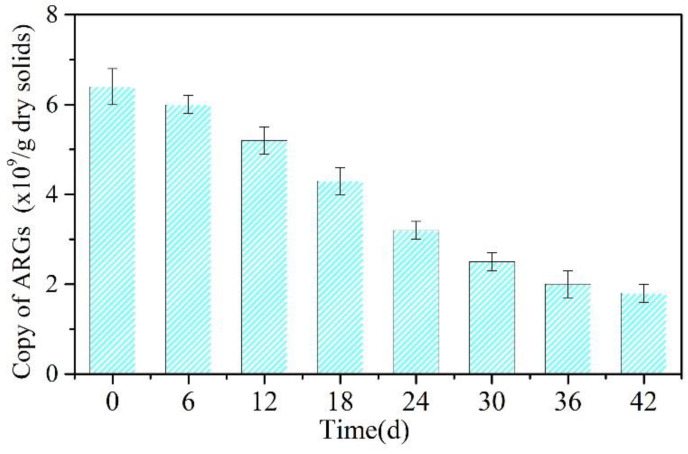
Rarefaction curve showing that the sequencing depth was sufficient to characterize the ARG profiles at the subtype level.

A total of 24 samples were acquired to analyze ARGs, which included raw excess dewatered sludge (RS) prior to MFC-Fenton treatment, OC, and samples obtained from MFC-Fenton reactors containing varying BES levels. Paired-end sequencing generated 686 ± 13 million raw reads (2 × 100 bp) across the 24 samples. These reads were merged into contigs (average length of 170 bp) using FLASH (v1.2.11), with a minimum overlap of 20 bp. In every sample, we subsampled tagged reads into 8,151,974 reads to compare all the samples. As revealed by the rarefaction curve (Figure 1), the samples were sequenced to an adequate depth for characterizing ARG subtypes.

The ARG levels within the sludge were reduced after the MFC-Fenton reaction (0, 0.1, 0.2, 0.3, 0.4, and 0.5 BES) and AnC (OC) for 42 days each (Figure S1). This finding aligns with prior research on composting [20]. At 0.5 g BES/g VSS, the mean ARG levels within the MFC-Fenton samples (0.5BES) were lower relative to the OC samples, suggesting that bioelectrochemically assisted AnC efficiently decreased the ARG levels within the sludge. Moreover, the resistome and microbiome structures were changed in the bioelectrochemical reactor samples, accompanied by persistently reduced overall, functional, and phylogenetic ARG diversity as well as network complexity [12]. The mean ARG levels in the MFC-Fenton samples with 0.1, 0.2, 0.3, 0.4, and 0.5 BES were lower relative to the MFC-Fenton samples with no BES (0BES) (ANOVA, *p* < 0.05). Therefore, the addition of BES significantly enhanced ARG attenuation within the MFC-Fenton system.

**Figure 2:**
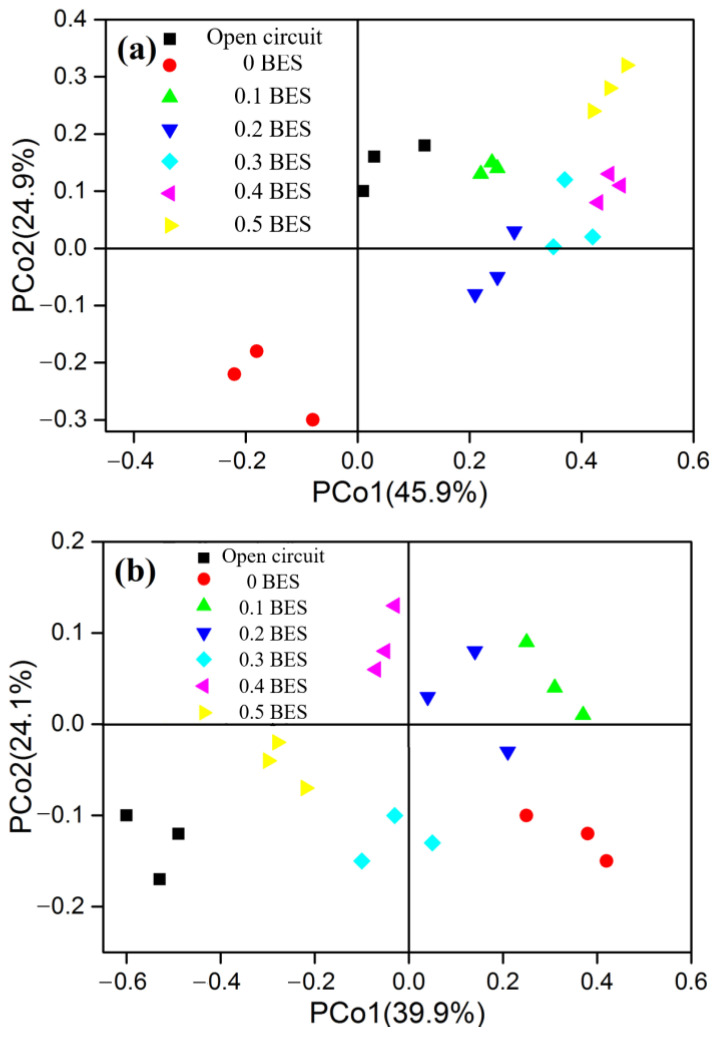
The variations in the absolute number of gene copies of ARGs in 0-0.5 BES on (**a**) day 0 and (**b**) day 42.

Figure S2 quantifies the ARG types and subtypes in each sample. There were 18 ARG types in the 24 MFC-Fenton samples. The detection of multiple ARG types in MFC-Fenton reactors is typically due to the many antibiotics being widely applied in veterinary and human medicine [4]. Of these 18 ARG types, ARGs related to aminoglycoside, bacitracin, tetracycline, and macrolide–lincosamide–streptogramin (MLS) could be detected in each sample, suggesting that they were very common inside the MFC-Fenton reactors. In addition, they were present in environmental samples (such as soils or human feces) [28]. It is worth noting that the levels of ARGs related to MLS and tetracycline increased in the BES-free MFC-Fenton samples, contributing to the increased overall ARG levels in the BES-free MFC-Fenton samples compared with the samples with BES (Figure S2). Adding 0.5 g BES/g VSS into the MFC-Fenton reactor reduced the levels of ARGs related to acriflavine, bleomycin, and fosfomycin in the OC MFC-Fenton system relative to the closed-circuit MFC-Fenton system. Based on the changes in the absolute number of ARG copies in 0.5BES throughout the 42-day period (Figure 2), the ARG reduction rates began increasing on day 24, reflecting a large decrease in ARB. Thereafter, the reduction rate slightly slowed.

#### 2.1.2. ARG Subtype Levels and Diversity

According to ARG type variations inside the MFC-Fenton reactors, we divided the ARG types into ARG subtypes. The acriflavine resistance genes encode the components of the multi-drug efflux system. This system serves to protect bacteria from hydrophobic inhibitors by actively transporting them out of the bacterial cell [29]. *Acr*B encodes the transporter protein that is stimulated by the proton-motive force, and it displays a broad substrate specificity compared to other known multidrug pumps. The *Acr*B level was higher in the OC MFC-Fenton system relative to the close-circuit MFC-Fenton system.

Aminoglycoside genes are widely distributed, which may be tightly associated with the wide application of aminoglycosides in treating infections. The major mechanism used by these ARGs is directly deactivating aminoglycosides [30]. There were 11 ARG subtypes that were detected in many samples. Among these 11 subtypes, the level of aminoglycoside ARGs markedly declined as the BES content in the MFC-Fenton system increased. Phosphotransferase is responsible for directly deactivating aminoglycosides, and this is the main mechanism underlying aminoglycoside resistance [2].

Meanwhile, four widely distributed bacitracin resistance gene subtypes were detected. Among them, two subtypes (Undecaprenyl Pyrophosphate Phosphatase and PGP Phosphatase), which enable the persistent synthesis of peptidoglycan and additional cell wall polymers in response to environmental stress [31], showed increased levels relative to the other subtypes (Figure S2b). There were three antibiotics in the MLS category, with most MLS-resistant subtypes detected [32]. Among these, the abundance of four subtypes (*lin*B, *mac*B, macrolide-efflux protein, and macrolide transporter ATP-binding protein) markedly decreased as the BES content increased. Lincosamide nucleotidyltransferase induces resistance to lincosamides but not to macrolides. Moreover, the other three widely distributed subtypes confer macrolide resistance through efflux pump resistance mechanisms [33].

Tetracyclines are broad-spectrum antibiotics that have been frequently utilized in animals and humans, with tetracycline resistance genes being commonly seen in various environmental samples [30]. The tetracycline resistance genes *tet*M, *tet*T, and *tet*W showed decreasing levels as the BES content increased from 0 to 0.5 gBES/g VSS (Figure S2b). The *tet*M, *tet*T and *tet*W genes encode for ribosomal protection proteins that modify tetracycline targets, conferring tetracycline resistance [34], while *tet*W shows a high specificity for anaerobic bacteria. Consequently, BES not only efficiently reduces methanogenesis, but can also reduce the levels of the *tet* genes that encode ribosomal protection proteins that modify tetracycline targets in bioelectrochemically assisted AnC.

The different shifts in ARG subtypes reflect distinct microbial adaptations to operational stressors. The increase in *acrB*-mediated acriflavine resistance under open-circuit conditions is likely a response to an accumulation of toxic intermediates when efflux pumps can enhance survival. Conversely, the declining aminoglycoside, MLS, and tetracycline resistance gene (*aph*, *tetM/T/W*, and *macB*) levels with increasing BES indicate an alleviation of antibiotic selection pressures. By inhibiting methanogens, BES disrupts syntrophic networks, reducing the competitive advantage of bacteria harboring these ARGs. Critically, the suppression of ribosomal protection proteins (*tetM/W/T*) and antibiotic-inactivating enzymes (*aph*) implies a reduced reservoir for horizontal transfer of high-risk resistance mechanisms. The increased bacitracin resistance further underscores that there is stress on cell-envelope maintenance processes. These shifts demonstrate that BES not only mitigates methanogenesis but indirectly attenuates clinically relevant ARGs in anaerobic systems, potentially lowering environmental resistome risks.

#### 2.1.3. Effect of BES on Reducing ARGs in MFC-Fenton System

**Figure 3:**
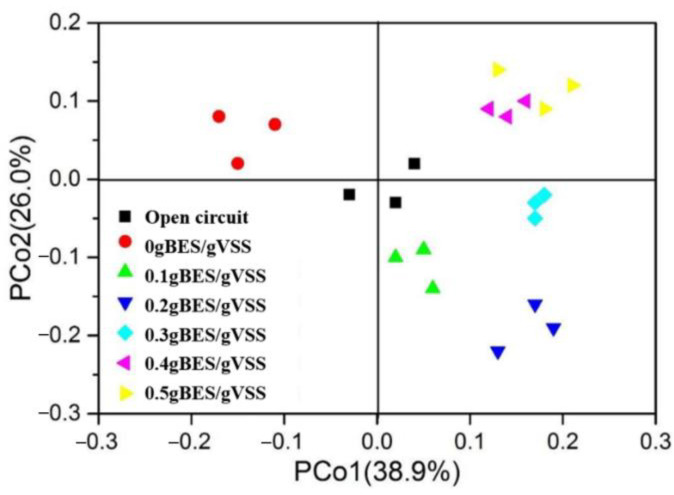
PCoA of all the samples based on ARG subtypes.

To determine how BES reduces ARGs in MFC-Fenton systems, we performed PCoA based on the Bray–Curtis distances of ARG subtypes (Figure 3). The BES-free MFC-Fenton samples obviously clustered apart from those of the OC MFC-Fenton system with BES, indicating that BES exerted a dominant effect on shaping the ARG profile compared to electrochemical factors alone. Therefore, the addition of BES could be an alternative approach to reduce ARGs [22]. Additionally, the OC and 0.5 g BES/g VSS closed-circuit MFC-Fenton system samples were significantly different. Thus, the introduction of BES into the anode chamber of the system influenced the ARG composition inside the MFC-Fenton reactors. Throughout the hydrolysis and biodegradation processes, extracellular DNA undergoes physical destruction, thus affecting the eventual ARG composition. Consequently, the decline in aminoglycoside, tetracycline, bacitracin, and MLS ARGs (Section 3.1) likely resulted from reduced host bacterial survival, as BES disrupted syntrophic networks and increased stress vulnerability.

This distinct ARG profile restructuring confirms BES’s unique dual mechanism: (1) disruption of syntrophic networks, reducing host bacteria carrying high-risk ARGs (e.g., ribosomal protection proteins), and (2) diminished functional plasmid availability (Section 3.2), limiting horizontal dissemination. Extracellular DNA degradation further erodes residual ARG reservoirs.

### 2.2. MGE Levels

Horizontal gene transfer (HGT) of bacteria- or environment-derived ARGs are mediated by MGEs, such as transposons, plasmids, integrons, and ISs [35]. Therefore, MGEs, namely plasmids, conjugative transposons, integrons, and ISs within the sludge metagenomes were analyzed.

Plasmid conjugation is believed to be the most common HGT method in nature. The lowest plasmid DNA level was detected in the MFC-Fenton system with 0.5 g BES/g VSS (0.170~0.200%) (Table S2). In the OC system, the plasmid DNA level was 0.200~0.230%. Thus, adding BES negatively affected plasmid DNA levels in the sludge (*p* < 0.05). BES facilitates sludge floc break-up and membrane lipid saponification [36], releasing plasmids into the extracellular environment. This disruption simultaneously depletes the intact host bacteria essential for conjugation and exposes plasmids to enzymatic degradation, ultimately reducing functional plasmid availability for horizontal transfer. According to Guo et al., a higher level of transferable plasmids increased the transfer frequency, resulting in more transconjugants [5]. Conjugative transposons (such as conjugative plasmids) possess both a transfer origin and the genes necessary for making the conjugation apparatus. When antibiotic resistance genes (ARGs) are present on conjugative or mobilizable plasmids, they can be transferred between different pathogens. Moreover, the specific resistance traits associated with these genes may disseminate throughout the entire ecosystem. From the metagenomic data, there were 3861–4687 reads (accounting for 0.114–0.151%) in the RS samples and 8048–9078 (0.371–0.425%) in the 0.5 BES samples; in contrast, in the OC samples, 11,876–14,167 reads (accounting for 0.508–0.542%) were matched with conjugative transposons (Table S2). This attenuation might influence the HGT rate of the sludge that is disposed of later.

ISs, which are DNA segments, are capable of transferring from one position on one chromosome to another position or chromosome. The lowest IS levels were detected within the 0.5 g BES/g VSS sludge (0.159–0.221% compared to 0.210–0.240% in the OC sludge) (Table S3), revealing the role of bioelectrochemical assistance in facilitating IS transfer for ARGs. In total, 189–308 reads (0.009–0.021%) in the 0.5 BES samples and 389–617 (0.012–0.035%) in the OC sludge were integrase genes. Among these, *intI* had the highest level of integrase genes, which accounted for 99.3–99.8% and 99.6–99.7% of the alignment hits for the 0.5 BES-treated and OC sludges, respectively. Integrase genes are frequently seen within WWTPs, such as class 1 integrons that carry different ARGs [1]. The reduced *tet*W level probably led to the strong relationship between MGEs.

When BES was added to the ES, the fermentation shifted toward alkaline anaerobic conditions, potentially restricting ARG transfer by reducing the abundance of genetic vectors, particularly plasmids and insertion sequences [37]. Critically, by disrupting syntrophic partnerships through methanogen inhibition, BES likely reduces plasmid-borne ARG dissemination in two key ways: (1) depletion of host bacteria carrying conjugative plasmids, and (2) impaired energy metabolism for plasmid replication and transfer under a lower redox potential. This aligns with the observed declines in MGE-linked ARGs (e.g., *tet* and MLS efflux pumps). Additionally, CF’s porous structure may decrease microbial contact and conjugation rates [38]. The thermophilic digestion reduced *tet* genes and *intI1*, while BES rapidly lowered the system redox potential—a critical electron source for microbial metabolism—thereby promoting ARG reduction. Thus, reactor performance is intrinsically linked to BES-driven redox shifts.

### 2.3. Relationships Between ARGs, MGEs, and MRGs

The changes in the concentration and types of ARGs are tightly linked to MGEs and MRGs [6,39]. These relationships were explored in the MFC-Fenton systems to reveal the effects of the different types of MRGs and MGEs on ARG reduction after BES addition and MFC-Fenton system processing.

#### 2.3.1. Association Between ARG Types and MRGs

**Figure 4:**
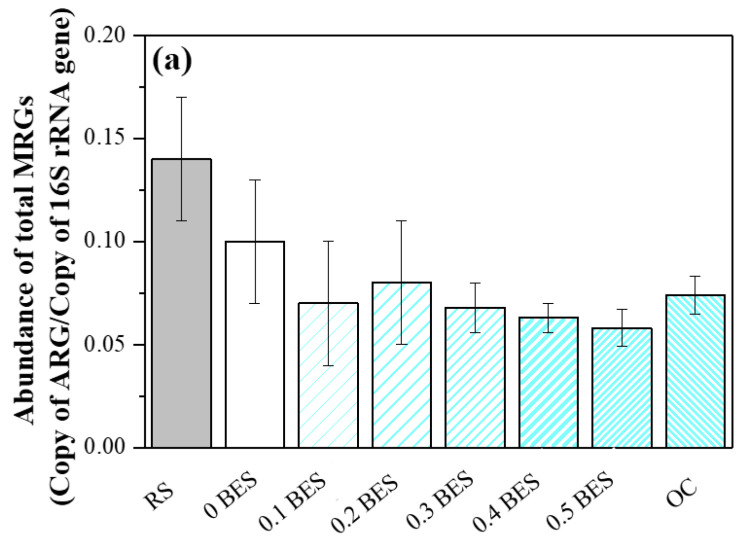

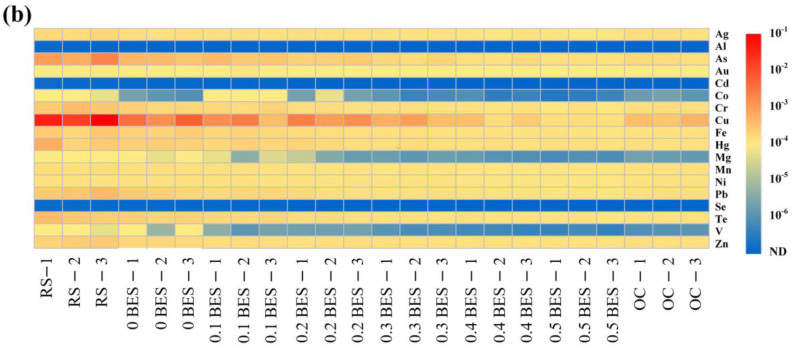
The abundances (**a**) and profiles (**b**) of MRGs in each sample.

**Figure 5:**
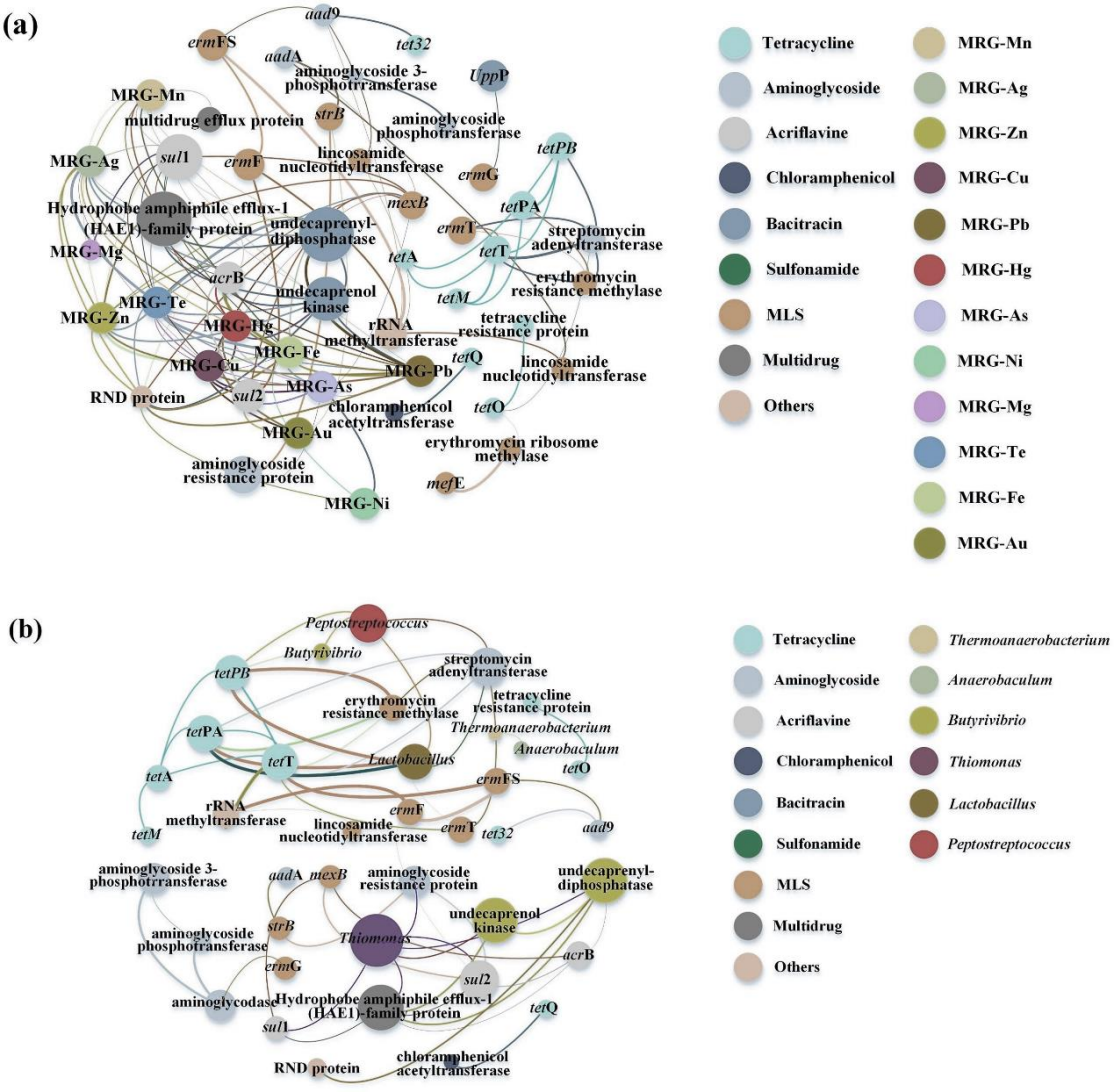
Network analysis revealing the co-occurrence patterns of ARG subtypes with MRGs (**a**) and microbial taxa (**b**).

The PCoA results (Figure S3a) showed that the samples with BES clustered together. To investigate if ARGs are related to MRGs, we conducted Procrustes analysis to determine if ARG levels correlated with MRG levels in each sample. Regardless of the slight differences in ARG and MRG compositions (Figure 4) and the dispersed distribution of the samples in the PCoA plot, the Procrustes analysis results suggested that the ARG composition was significantly related to the MRG composition (*p* = 0.001) (Figure S3a); however, it did not show the precise associations of certain ARG subtypes with MRGs. A network analysis suggested that ARG subtypes co-occurred with MRGs; these results depict the relationships with high resolution (Figure 5). For instance, *sul*1 coincided with resistance genes related to Cu, Fe, Hg, Mg, Mn, etc., whereas *sul*2 coincided with those associated with As, Hg, and Te. Such findings confirmed the prior results and suggest the co-occurrence of *sul*1 and *sul*2.

The ARG and MRG co-occurrence revealed the potential co-selection ability of the MFC-Fenton system. Moreover, indirect responses to antibiotic and metal exposures are one of the co-selection mechanisms of prokaryotes [40]. For example, the physically connected *str*B and Hg resistance genes in the pHCM1 plasmid are a representative example of resistance [28]. Moreover, the connection between *mex* and *czc* operons may induce metal efflux and imipenem resistance. According to our findings, *mex*B co-occurred with ARGs for Ag and Cu, suggesting a different regulatory control system for the transcriptional linkage.

#### 2.3.2. Association Between Microbial Community Abundance and ARGs

In Section 2.1, the addition of BES and use of an MFC-Fenton system were found to significantly affect the ARG composition. The restriction of the host range is likely crucial for reducing ARGs inside an MFC-Fenton system. Therefore, the microbial community composition in each sample was analyzed (Table S4) and PCoA was performed to describe the variations and distances between samples (Figure S5a). Different from the PCoA results for the ARG composition, the microbial community composition in the MFC-Fenton samples obviously clustered apart from that of the OC samples. The MFC-Fenton samples with or without BES addition clustered together in contrast to the PCoA results for ARG composition. This was due to the diverse distributions of genera associated with extracellular electron transfer (*Geobacter*, *Desulfobulbus*, *Desulfotomaculum*, and *Aeromonas*) in the MFC-Fenton and OC samples [41]. The bacteria involved in hydrolysis, electrogenesis, and acidogenesis were enriched in the MFC-Fenton system following BES addition. The enrichment of key functional taxa after BES treatment directly explains the system improvements: *Saprospiraceae* enhanced sludge hydrolysis, increasing substrate availability for electrogens; *Geobacter* and *Anaerolineae* boosted electrogenesis, correlating with a 1.7-fold higher power density [42]; and *Clostridia* dominated acidogenesis, driving VFA accumulation that inhibits ARG hosts. This functional restructuring underpins BES’s dual functions in waste valorization and resistome mitigation [43].

To investigate if ARGs are related to the microbial composition, we conducted Procrustes analysis on the ARG levels and microbial community in each sample. ARG level was significantly correlated with bacterial community (M^2^ = 0.2876) if each sample was considered. Consequently, the overall composition and abundance of the biological community was the main contributor to ARG diversity within the MFC-Fenton systems. A prior study also came to the same conclusion, suggesting that alterations in the microbial community might be the major cause of diverse ARGs [27]. Therefore, the BES addition and bioelectrochemical assistance influenced the ARG levels and microbial community composition. The operating conditions may also be major factors for bacterial community distribution and ARG spread, and the biological community with ARGs utilizing various resistance mechanisms have different reactions under different environmental conditions.

**Figure 6:**
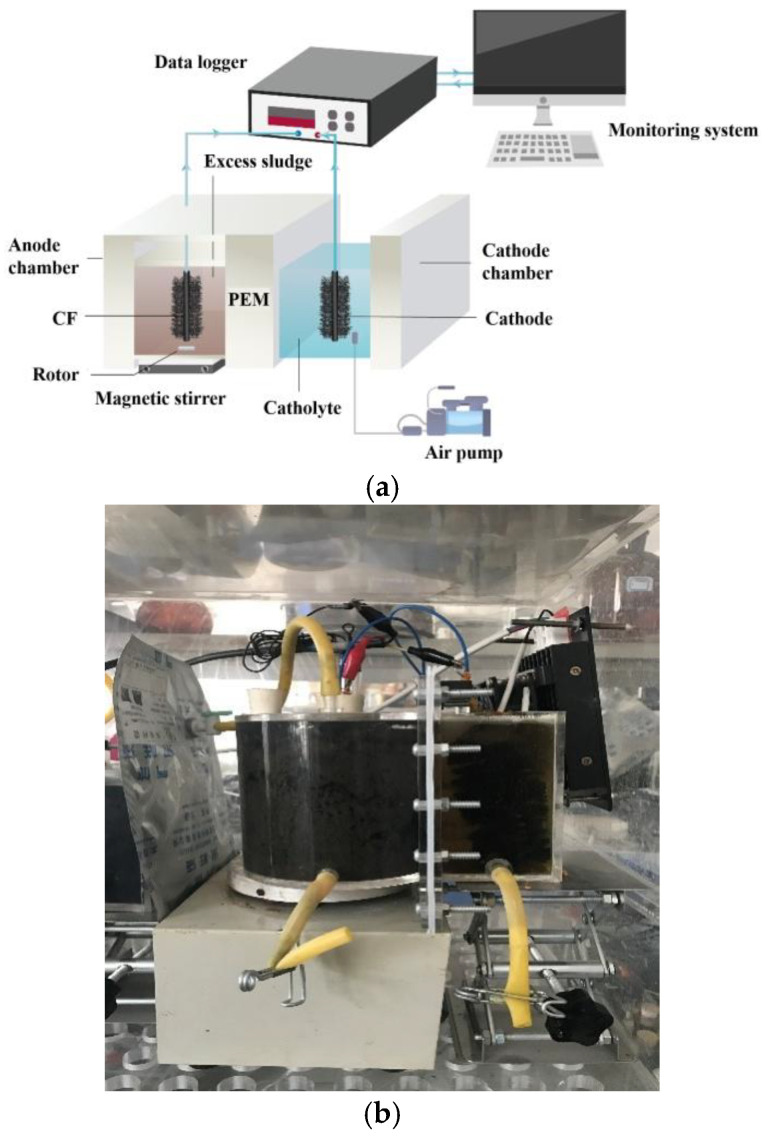
Schematic diagram (**a**) and picture of reactor (**b**) of the MFC-Fenton system.

The Procrustes analysis shed light on the associations between ARGs and the microbial community composition within MFC-Fenton systems (Figure 4b). However, it could not identify the precise associations between specific ARG subtypes and microbial taxa. Network analysis (Figure 6b) was performed to illustrate their co-occurrence and provide a valuable complement to the overall relationship data. The network analysis identified *Thiomonas* as a high-risk ARG reservoir (linking *sul1*, *sul2*, and *mexB*). Crucially, its lower levels in BES-treated sludge (Table S5) indicates reduced sulfonamide resistance (Figure 1), suggesting that (1) Thiomonas’s sulfur-metabolizing niche favors sul gene maintenance and (2) BES disrupts this niche through syntrophic partner inhibition (Section 3.2). Similar host–ARG co-occurrences in *Peptostreptococcus-tetPB* further confirmed this taxon-specific resistance carryover.

#### 2.3.3. Relationships Between ARGs, MGEs, and MRGs

RDA was conducted to analyze the relationships between microorganism species, ARGs, MGEs, and MRGs in the MFC-Fenton system (Figure S5). These chosen variables were found to account for 86.9% of the factors that affected ARG levels. Such findings reflect the diverse features observed in composting. *Firmicutes* was dominant in the early stage of the process, whereas MGEs were dominant as the ARG level increased after obtaining nutrients, and microorganisms showed higher activity in the cooling stage. During the maturation stage, *Actinobacteria* had the greatest contributions to the changes in ARG levels (62.8%). To identify the most important contributing factors for ARG changes and to separate the functions of microorganism species, variables and covariates were chosen for the RDA. The main contributors were found to be (in descending order) bacterial community, MGEs, and MRGs (Figure S5). Therefore, microorganism species variations significantly influenced the ARG variations but not co-selection of ARGs via HGT or with MRGs through MGEs. Similar findings have been reported under diverse environmental conditions, such as rivers, WWTPs, and soils [35,44]. MGEs had a smaller contribution, suggesting that HGT might be insufficient to influence the association between ARGs and the bacterial community in MFC-Fenton processes.

In the ES compost, these chosen variables accounted for over 79.3% of the variation in species carrying antibiotics resistance genes, with *Firmicutes* showing a positive correlation and accounting for 13.7% of the overall environment. MRGs accounted for 14.6% of the variance, which is lower than the 16.2% explained by MGEs. This finding is consistent with a study on swine manure co-composted with Chinese medicinal herbal residues, which found that microorganism species were the main contributor to variance, while MGEs accounted for 4.1% [35]. In another study, environmental factors explained 92.7% of the overall ARG and MGE variance in swine manure composting [39]. In this study, the variance partitioning analysis (RDA-based) confirmed the microbial community as the primary driver of ARG distribution in soils (63.7%, *p* = 0.002 according to Monte Carlo permutation test), significantly surpassing MGEs (15.7%, *p* = 0.038) and MRGs (13.2%, *p* = 0.045) in explanatory power.

## 3. Materials and Methods

### 3.1. MFC-Fenton System Configuration and Operation

The dual-chamber MFC-Fenton reactor was configured as shown in Figure 6: a 550-mL anode chamber was prepared and assembled into a sealed cylinder configuration (Φ 85 mm × 100 mm), where a reference electrode (Ag/AgCl, +0.195 V vs. SHE) and gas collection bag were located on top, and a magnetic stirring apparatus (JB-2, Shanghai Leici, Shanghai, China) was on the bottom, thus ensuring a uniform ES. Meanwhile, a 360 mL cathode chamber was prepared in an unsealed cuboid configuration (60 mm × 60 mm × 100 mm) with a proton exchange membrane (PEM, Nafion 117, DuPont, Wilmington, DE, USA). In the electrode, carbon felt (CF) served as the cathode and anode material during the start-up stage, while copper wires were used to connect electrodes with the external resistance (1000 Ω).

During the startup stage, ES (Table S1) and a nutrient solution were added to the anode chamber and potassium ferricyanide (32.9 g/L) was used as the electron acceptor in the cathode chamber inside the MFC-Fenton reactors. We utilized the same nutrient solution as the one used in our prior research [45]. The stable output voltage was deemed to be an indicator of successful startup of the reactors. The systems reached a stable state in about 15 days. After initiation, fresh ES was added into the anodic chambers inside the reactors and the solution in the cathode chamber was replaced with deionized water. BES solution with varying doses (0, 0.1, 0.2, 0.3, 0.4, and 0.5 g BES/g VSS) were prepared, with the reactors being denoted as 0, 0.1, 0.2, 0.3, 0.4, and 0.5 BES. The same ES was used in the control, and 0.5 g BES/g VSS was added in the open-circuit system, which was denoted as OC. Each reaction was operated with no voltage control for 40 days. Every experiment was performed in the fed-batch mode at 25 ± 2 °C. During this process, sludge samples from the anode chamber were taken for testing, with each sample being analyzed thrice. During the 40 days of system startup and operation, no new sludge was added to the systems.

### 3.2. Metagenomic Sequencing and DNA Isolation

After 40 days of operation, samples were collected from each reactor and delivered to the laboratory within 24 h. Total genomic DNA was isolated from each sample using the Power Soil DNA Isolation Kit (Mo Bio, Carlsbad, CA, USA). The DNA content and quality were measured using an Epoch Microvolume Spectrophotometer (BioTek, Winooski, VT, USA). The genomic DNA was transported to Shanghai Meiji Biological Company (Shanghai, China) where libraries were established and sequencing was conducted using an Illumina HiSeq 2000 platform.

### 3.3. ARG, MGE, and MRG Analyses

To analyze ARGs, BLASTX was utilized to blast the metagenomic tags against the ARG Comprehensive Antibiotic Resistance Database (CARD, version 3.2.5). If the optimal hit of one tagged read was >90% similar to an ARG reference sequence, this read would be deemed an ARG-like sequence. All ARG-like sequences were classified as diverse ARG types and subtypes using customized scripts, with their levels were standardized based on the lengths of the 16S rRNA gene and ARG reference sequences. To analyze MGEs, integrase gene, IS, and plasmid sequences were downloaded from the INTEGRALL (http://integrall.bio.ua.pt/, accessed on 13 May 2025), ISfineder (https://www-is.biotoul.fr/, accessed on 15 May 2025), and NCBI plasmid genome databases to create local integron, plasmid, and IS databases. The sequences were aligned to three MGE databases using BLASTX to identify plasmid-, IS-, and integron-like sequences. In the MRG analysis, we blasted the metagenomic tags against the MRG BacMet database (version 2.0). If the optimal hit of one tagged read was >90% similar to an MRG reference sequence, this read would be deemed as an MRG-like sequence. MRG levels were standardized based on the lengths of the 16SrRNA gene and MRG reference sequences.

### 3.4. Microbial Community Analyses

To investigate the bacterial and methanogenic community structures, the V3–V4 region of the bacterial 16S rRNA gene was amplified using the universal primers 341F (5′-CCCTACACGACGCTCTTCCGATCTG-3′) and 805R (5′-GACTGGAGTTCCTTGGCACCCGAGAATTCCA-3′). The resulting paired-end sequences were processed using the QIIME2 pipeline (version 2023.7). Raw reads were quality-filtered, denoised, and merged using DADA2 to generate amplicon sequence variants (ASVs). Taxonomic classification of ASVs was performed using the Silva SSU rRNA database (release 138.1) with a naive Bayes classifier at a confidence threshold of 0.8.

All chemical analyses were performed following the standard methods mentioned in our previous study [20,27,46]. Each statistical test was conducted thrice, with *p <* 0.05 considered statistically significant. This study used three identical reactors operating simultaneously, and the collected samples were mixed for testing, which to a certain extent, reduced the variability in the results. To determine the contributions made by MGEs and bacterial communities to ARGs, the “pheatmap”, “vegan”, and “MASS” packages of R (Version 4.3.2) were utilized for the heatmap and variation partitioning analyses. Meanwhile, based on Bray–Curtis distance, ARG, MRG, MGE, and bacterial community patterns were assessed through principal coordinate analysis (PCoA), while correlations among them were analyzed using the Mantel test and redundancy analysis (RDA). Canoco version 5.0 software was used to conduct PCoA and RDA, while Cytoscape 3.3.0 was used to display the network using a circular layout algorithm.

## 4. Conclusions

Under the tested conditions (0–0.5 g BES/g VSS), 2-bromoethane sulfonate (BES) attenuated ARG levels during bioelectrochemically assisted excess sludge anaerobic digestion. Lincosamide nucleotidyltransferase, *mac*B, macrolide-efflux protein, and macrolide transporter ATP-binding protein ARG levels decreased as the BES dose increased. Regarding ARG reduction and cost-effectiveness, 0.4 g BES/g VSS was sufficient in this MFC-Fenton system. Moreover, BES induced low-ratio methanogenic bacterial conditions and the extracellular electron transfer-induced bioelectrochemical assistance promoted extracellular DNA decomposition, thus affecting the eventual ARG composition. The MGE levels decreased after BES addition and bioelectrochemical assistance, thereby influencing the HGT rates of the sludge after treatment. Bacterial community contributed the most to the ARG variation (63.7%), with *Actinobacteria* and *Firmicutes* accounting for 52.8% of the variation. Furthermore, the *sulI*, *sulII*, *tet*G, and *bla*_TEM_ levels positively correlated with the MRG levels, and they decreased in the anode chamber following the MFC-Fenton reaction.

## Data Availability

The raw data supporting the conclusions of this article will be made available by the authors on request.

## Supplementary Materials

The following supporting information can be downloaded at: https://www.mdpi.com/article/10.3390/molecules30173502/s1, Figure S1: Abundances of ARGs in each sample and in five groups; Figure S2: Broad-spectrum profile of the ARG types (number of copies of ARG per copy of 16S rRNA gene). (a) Abundances of the 100 major (>2.0 × 10^–4^ copies of ARG per copy of 16S rRNA gene in at least one sample) ARG subtypes in MFC-Fenton samples; Figure S3: Procrustes analyses of ARGs and MRGs (a) and microbial community (b). Figure S4: PCoA of all the samples based on MRG composition (a) and microbial community (b); Figure S5: RDA of all the samples based on ARG subtype (a) and variation partitioning analysis on the effects of the bacterial community, MGEs, and MRGs on the ARG abundances (b); Table S1: The basic chemical characteristic parameters of the excess sludge; Table S2: Summary of MGE abundances, including the abundances of plasmids, conjugative transposons, ISs, and integrons, in the 24 sludge metagenomes; Table S3: DNA yield and metagenomics sequencing results; Table S4: Microbial community profiles in samples; Table S5: Potential ARG hosts revealed by co-occurrence between ARG subtypes and microbial taxa [47,48].

## List of Abbreviations

AnC, anaerobic composting; ARG, antibiotic resistance gene; ARB, antibiotic resistant bacteria; BES, 2-bromoethane sulfonate; CF, carbon felt; CoM, coenzyme M; CoB, coenzyme B; CE, coulombic efficiency; ES, excess dewatered sludge; Fe^0^, zero-valent iron; HGT, horizontal gene transfer; MFC-Fenton, MFC-powered Fenton system; MRGs, metal resistance genes; MGEs, mobile genetic elements; MLS, macrolide–lincosamide–streptogramin; PCoA, principal coordinate analysis; RDA, redundancy analysis; RS, raw excess dewatered sludge.

## References

[1] Qian X., Gunturu S., Guo J., Chai B., Cole J.R., Gu J., Tiedje J.M. (2021). Metagenomic analysis reveals the shared and distinct features of the soil resistome across tundra, temperate prairie, and tropical ecosystems. Microbiome.

[2] Zhang B., Yang R., Liu Y., Guo J., Yang J., Qin X., Wang S., Liu J., Yang X., Zhang W. (2025). From glacier forelands to human settlements: Patterns, environmental drivers, and risks of antibiotic resistance genes. J. Hazard. Mater..

[3] Zhao X., Wang Z., Xu T., Feng Z., Liu J., Luo L., He Y., Xiao Y., Peng H., Zhang Y. (2021). The fate of antibiotic resistance genes and their influential factors during excess sludge composting in a full-scale plant. Bioresour. Technol..

[4] Tian Z., Chi Y., Yu B., Yang M., Zhang Y. (2019). Thermophilic anaerobic digestion reduces ARGs in excess sludge even under high oxytetracycline concentrations. Chemosphere.

[5] Guo H., Li Z., Sun X., Xing M. (2024). Impact of earthworms on suppressing dissemination of antibiotic resistance genes during vermicomposting treatment of excess sludge. Bioresour. Technol..

[6] Yu H., Zhao Q., Meng F., Ruan L., Sun T., Liu X., Liu W., Zhu Y., Li W., Meng F. (2020). Deciphering the role of calcium peroxide on the fate of antibiotic resistance genes and mobile genetic elements during bioelectrochemically-assisted anaerobic composting of excess dewatered sludge. Chem. Eng. J..

[7] Callegari A., Tucci M., Aulenta F., Cruz Viggi C., Capodaglio A.G. (2025). Anaerobic sludge digestion enhancement with bioelectrochemical and electrically conductive materials augmentation: A state of the art review. Chemosphere.

[8] Feng R., Wang C., Li Y., Huang J., Wang Y. (2025). Occurrence, fate, effects and control of coagulants/flocculants in anaerobic digestion of waste activated sludge: A review. J. Water Process Eng..

[9] Olvera-Vargas H., Zheng X., Garcia-Rodriguez O., Lefebvre O. (2019). Sequential “electrochemical peroxidation-Electro-Fenton” process for anaerobic sludge treatment. Water Res..

[10] Jiang J.Q., Zhao Q.L., Wang K., Wei L.L., Zhang G.D., Zhang J.N. (2010). Effect of ultrasonic and alkaline pretreatment on sludge degradation and electricity generation by microbial fuel cell. Water Sci. Technol..

[11] Luo L., Wang G., Wang Z., Ma J., He Y., He J., Wang L., Liu Y., Xiao H., Xiao Y. (2021). Optimization of Fenton process on removing antibiotic resistance genes from excess sludge by single-factor experiment and response surface methodology. Sci. Total Env..

[12] Li H., Xu H., Song H.L., Lu Y., Yang X.L. (2020). Antibiotic resistance genes, bacterial communities, and functions in constructed wetland-microbial fuel cells: Responses to the co-stresses of antibiotics and zinc. Environ. Pollut..

[13] Xia H., Yang J., Huang K., Nie C. (2023). Microplastics into vermi-wetland lower the treatment performance of organic substances and antibiotic resistance genes in excess sludge. J. Environ. Chem. Eng..

[14] Wang H., Luo H., Fallgren P.H., Jin S., Ren Z.J. (2015). Bioelectrochemical system platform for sustainable environmental remediation and energy generation. Biotechnol. Adv..

[15] Zhang L., Fu G., Zhang Z. (2020). Long-term stable and energy-neutral mixed biofilm electrode for complete nitrogen removal from high-salinity wastewater: Mechanism and microbial community. Bioresour. Technol..

[16] Wang Y.T., Wang R.S. (2017). A Bio-Electro-Fenton System Employing the Composite FePc/CNT/SS316 Cathode. Material.

[17] Su C., Lu Y., Deng Q., Chen S., Pang G., Chen W., Chen M., Huang Z. (2019). Performance of a novel ABR-bioelectricity-Fenton coupling reactor for treating traditional Chinese medicine wastewater containing catechol. Ecotoxicol. Environ. Saf..

[18] Rago L., Guerrero J., Baeza J.A., Guisasola A. (2015). 2-Bromoethanesulfonate degradation in bioelectrochemical systems. Bioelectrochemistry.

[19] Zhao H., Kong C.H. (2018). Elimination of pyraclostrobin by simultaneous microbial degradation coupled with the Fenton process in microbial fuel cells and the microbial community. Bioresour. Technol..

[20] Wang W., Wang K., Zhao Q., Liu Y. (2022). Simultaneous degradation of anodic sludge and cathodic refractory pollutant in a MFC powered EF system enhanced by co-addition of lysozyme and 2-bromoethane sulfonate. J. Environ. Chem. Eng..

[21] Zhang Y., Jiang J., Zhao Q., Gao Y., Wang K., Ding J., Yu H., Yao Y. (2017). Accelerating anodic biofilms formation and electron transfer in microbial fuel cells: Role of anionic biosurfactants and mechanism. Bioelectrochemistry.

[22] Ondon B.S., Li S., Zhou Q., Li F. (2020). Simultaneous removal and high tolerance of norfloxacin with electricity generation in microbial fuel cell and its antibiotic resistance genes quantification. Bioresour. Technol..

[23] Wei L., Li J., Xue M., Wang S., Li Q., Qin K., Jiang J., Ding J., Zhao Q. (2019). Adsorption behaviors of Cu^2+^, Zn^2+^ and Cd^2+^ onto proteins, humic acid, and polysaccharides extracted from sludge EPS: Sorption properties and mechanisms. Bioresour. Technol..

[24] Zhang S., Song H.L., Cao X., Li H., Guo J., Yang X.L., Singh R.P., Liu S. (2019). Inhibition of methanogens decreased sulfadiazine removal and increased antibiotic resistance gene development in microbial fuel cells. Bioresour. Technol..

[25] Yin C., Shen Y., Yuan R., Zhu N., Yuan H., Lou Z. (2019). Sludge-based biochar-assisted thermophilic anaerobic digestion of waste-activated sludge in microbial electrolysis cell for methane production. Bioresour. Technol..

[26] Lu Y., Sun R., Zhang C., Ding S., Ying M., Shan S. (2021). In situ analysis of antibiotic resistance genes in anaerobically digested dairy manure and its subsequent disposal facilities. Bioresour. Technol..

[27] Wang W., Wang K., Zhao Q., Yang L. (2022). Maximizing electron flux, microbial diversity and gene abundance in MFC powered electro-Fenton system by optimizing co-addition of lysozyme and 2-bromoethanesulfonate. J. Env. Manag..

[28] Ejileugha C. (2022). Biochar can mitigate co-selection and control antibiotic resistant genes (ARGs) in compost and soil. Heliyon.

[29] Zainab S.M., Junaid M., Xu N., Malik R.N. (2020). Antibiotics and antibiotic resistant genes (ARGs) in groundwater: A global review on dissemination, sources, interactions, environmental and human health risks. Water Res..

[30] Wu D., Wang L., Su Y., Dolfing J., Xie B. (2021). Associations between human bacterial pathogens and ARGs are magnified in leachates as landfill ages. Chemosphere.

[31] Li S., Hua T., Yuan C.S., Li B., Zhu X., Li F. (2020). Degradation pathways, microbial community and electricity properties analysis of antibiotic sulfamethoxazole by bio-electro-Fenton system. Bioresour. Technol..

[32] Tian Z., Zhang Y., Yang M. (2018). Chronic impacts of oxytetracycline on mesophilic anaerobic digestion of excess sludge: Inhibition of hydrolytic acidification and enrichment of antibiotic resistome. Env. Pollut..

[33] Yu H., Jia W., Luo Y., Zhang R., Zhao J., Lu C., Dong Y., Shuo H., Li B., Qu C. (2025). Accelerating enrichment of ARGs and MGEs with increasing ammonium removal during partial nitrification treating high-strength ammonia wastewater. Env. Res..

[34] Ding J., Wei L., Huang H., Zhao Q., Hou W., Kabutey F.T., Yuan Y., Dionysiou D.D. (2018). Tertiary treatment of landfill leachate by an integrated Electro-Oxidation/Electro-Coagulation/Electro-Reduction process: Performance and mechanism. J. Hazard. Mater..

[35] Zhang S., Abbas M., Rehman M.U., Huang Y., Zhou R., Gong S., Yang H., Chen S., Wang M., Cheng A. (2020). Dissemination of antibiotic resistance genes (ARGs) via integrons in Escherichia coli: A risk to human health. Env. Pollut..

[36] Fang Z., Song H.L., Cang N., Li X.N. (2015). Electricity production from Azo dye wastewater using a microbial fuel cell coupled constructed wetland operating under different operating conditions. Biosens. Bioelectron..

[37] Mortezaei Y., Demirer G.N., Williams M.R. (2024). Fate of intracellular and extracellular antibiotic resistance genes in sewage sludge by full-scale anaerobic digestion. Sci. Total Env..

[38] Mousset E., Wang Z., Hammaker J., Lefebvre O. (2017). Electrocatalytic phenol degradation by a novel nanostructured carbon fiber brush cathode coated with graphene ink. Electrochim. Acta.

[39] Yang Y., Chen W., Yin J., Jiang T., Zhao L., Li G., Wang G., Yuan J. (2025). Interactions between fungi and bacteria hosts carrying MGEs is dominant for ARGs fate during manure mesophilic composting. Waste Manag..

[40] Shao B., Liu Z., Tang L., Liu Y., Liang Q., Wu T., Pan Y., Zhang X., Tan X., Yu J. (2022). The effects of biochar on antibiotic resistance genes (ARGs) removal during different environmental governance processes: A review. J. Hazard. Mater..

[41] Wang Y., Zhang H., Feng Y., Li B., Yu M., Xu X., Cai L. (2019). Bio-Electron-Fenton (BEF) process driven by sediment microbial fuel cells (SMFCs) for antibiotics desorption and degradation. Biosens. Bioelectron..

[42] Deng Q., Su C., Lu X., Chen W., Guan X., Chen S., Chen M. (2020). Performance and functional microbial communities of denitrification process of a novel MFC-granular sludge coupling system. Bioresour. Technol..

[43] Jung S., Regan J.M. (2011). Influence of external resistance on electrogenesis, methanogenesis, and anode prokaryotic communities in microbial fuel cells. Appl. Env. Microbiol..

[44] Wang J., Xu S., Zhao K., Song G., Zhao S., Liu R. (2023). Risk control of antibiotics, antibiotic resistance genes (ARGs) and antibiotic resistant bacteria (ARB) during sewage sludge treatment and disposal: A review. Sci. Total Env..

[45] Yu H., Jiang J., Zhao Q., Wang K., Zhang Y., Zheng Z., Hao X. (2015). Bioelectrochemically-assisted anaerobic composting process enhancing compost maturity of dewatered sludge with synchronous electricity generation. Bioresour. Technol..

[46] Wang L., Trujillo S., Liu H. (2019). Selective inhibition of methanogenesis by acetylene in single chamber microbial electrolysis cells. Bioresour. Technol..

[47] Costa B.F., Zarei-Baygi A., Md Iskander S., Smith A.L. (2023). Antibiotic resistance genes fate during food waste management-Comparison between thermal treatment, hyperthermophilic composting, and anaerobic membrane bioreactor. Bioresour. Technol..

[48] Kong L., Qi Y., Shi X. (2024). Variations in antibiotic resistance genes during long-term operation of an upflow anaerobic sludge blanket reactor. Environ. Res.

