# Supplementary material for: Reduction in ARGs and Mobile Genetic Elements Using 2-Bromoethane Sulfonate in an MFC-Powered Fenton System
Source: Molecules. 2025 Aug 26;30(17):3502. doi: 10.3390/molecules30173502 (PMC12430619; doi:10.3390/molecules30173502)
Supplement: Supplementary file 1 [file molecules-30-03502-s001.zip › molecules-3778077-supplementary.pdf]

Supplementary Materials

Revealing the contribution of 2-bromoethane sulfonate on the reduction of ARGs and mobile genetic elements in MFC powered Fenton system

Weiye Wang[a, b], Jian Wei[a, b*], Zhuang Guo[a,b], Xiaodong Bai[a], Yonghui Song[a, b*]

a State Key Laboratory of Environmental Criteria and Risk Assessment, Chinese Research Academy of Environmental Sciences, Beijing 100012, China

b Institute of Water Ecology and Environment, Chinese Research Academy of Environmental Sciences, Beijing 100012, China

[*] Corresponding author: Jian Wei

E-mail: weijian0911@163.com

Corresponding author: Yonghui Song

E-mail: songyh@craes.org.cn.

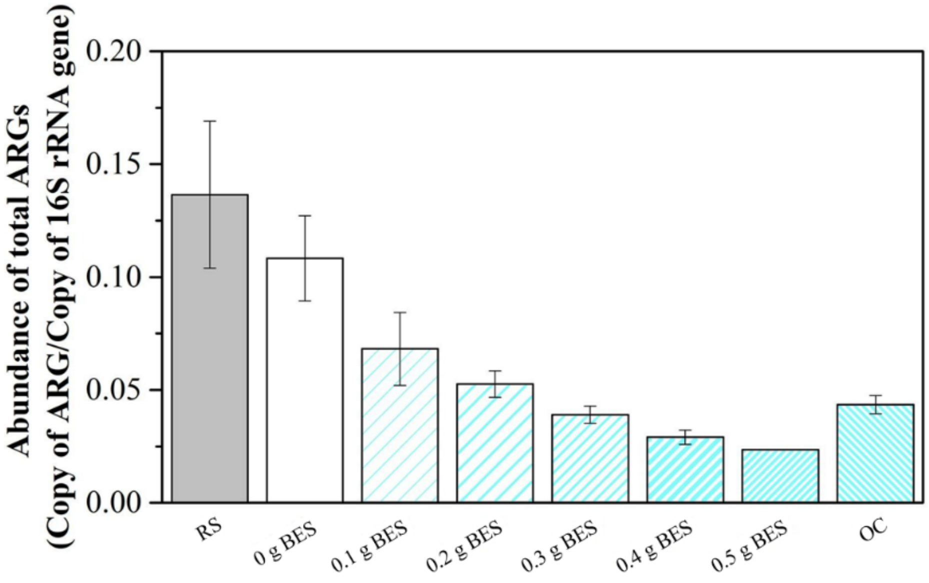

Fig. **S1.** Abundances of ARGs in each sample and in five groups.

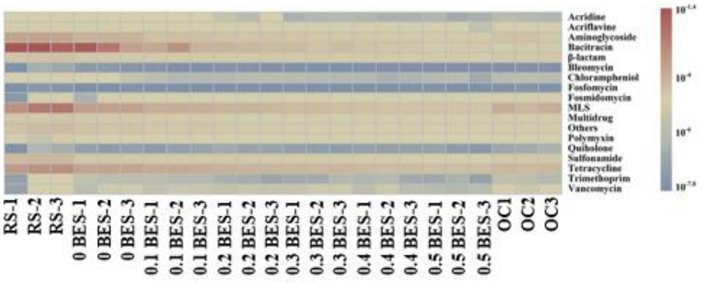

(a)

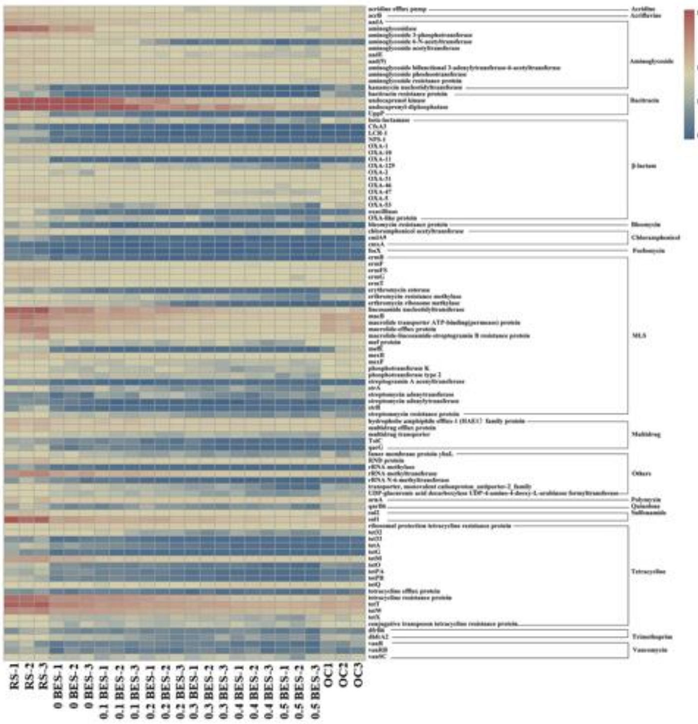

(b)

Fig. S2. Broad-spectrum profile of the ARG types (copy of ARG per copy of 16S-rRNA gene) (a); abundances of the 100 major (> 2.0×10^{-4} copy of ARG per copy of 16S-rRNA gene in at least one sample) ARG subtypes in MFC-Fenton samples.

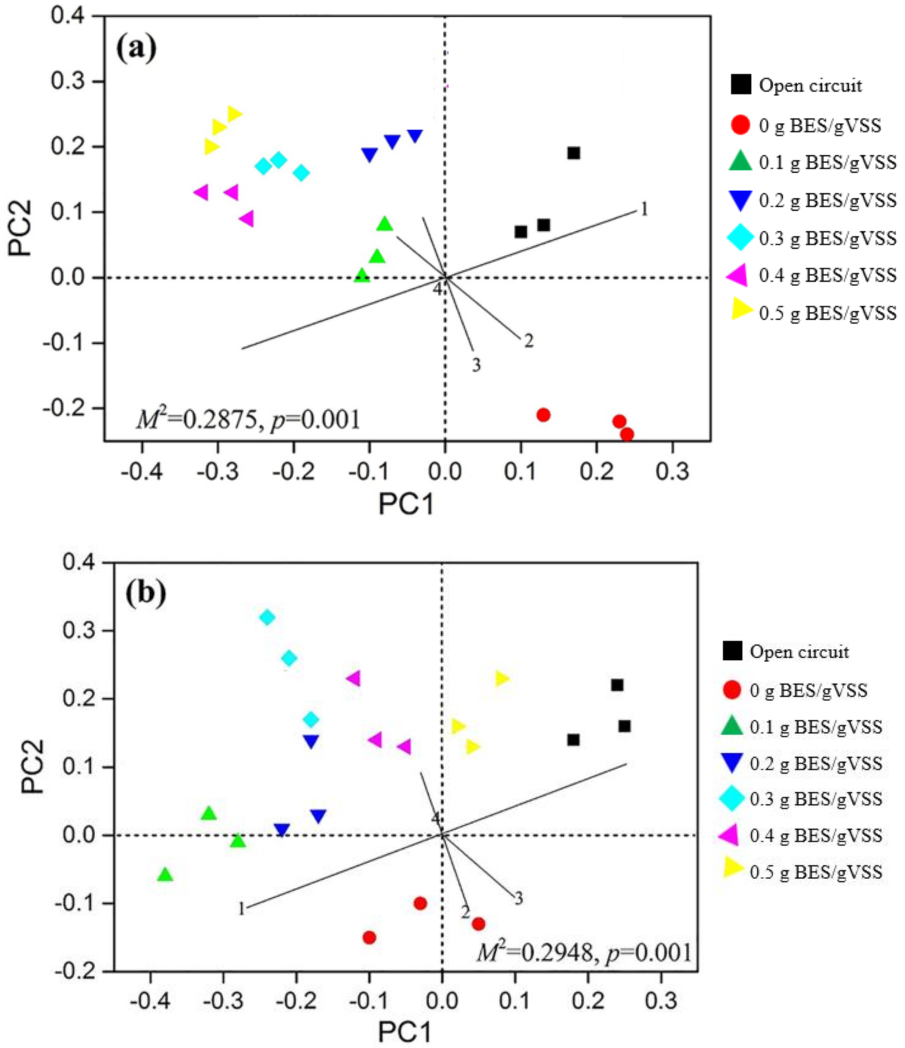

Fig. S3. Procrustes analyses of ARGs with MRGs (a) and microbial community (b).

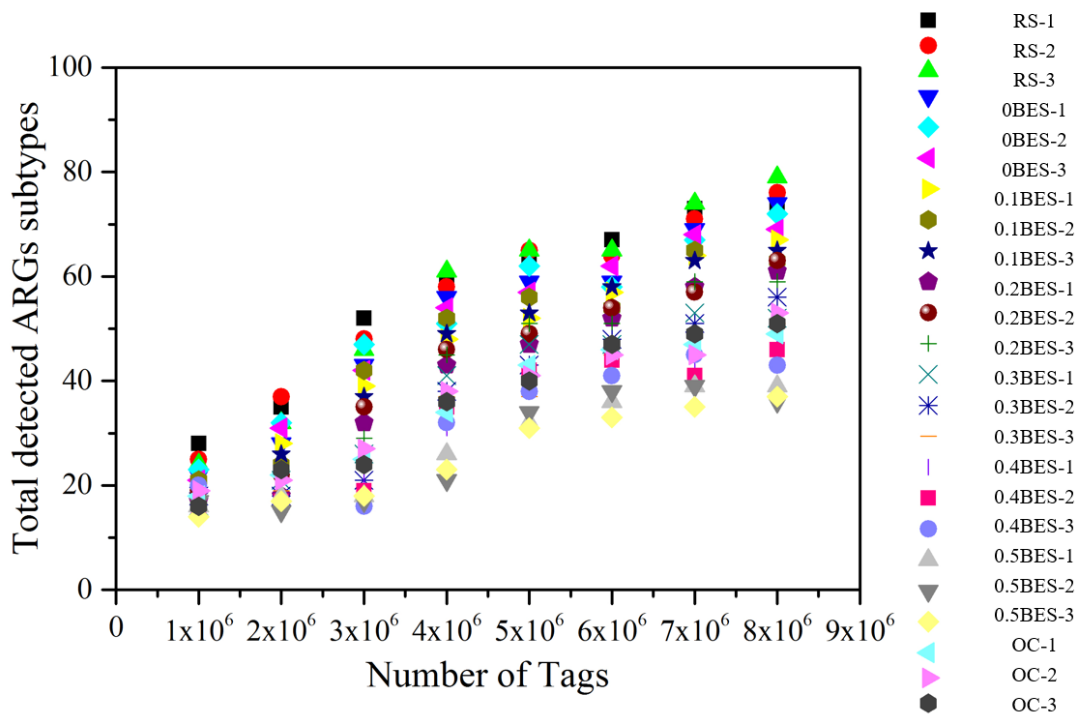

Fig. **S4** PCoA analysis of all the samples based on MRG compositions (a) and microbial community (b)

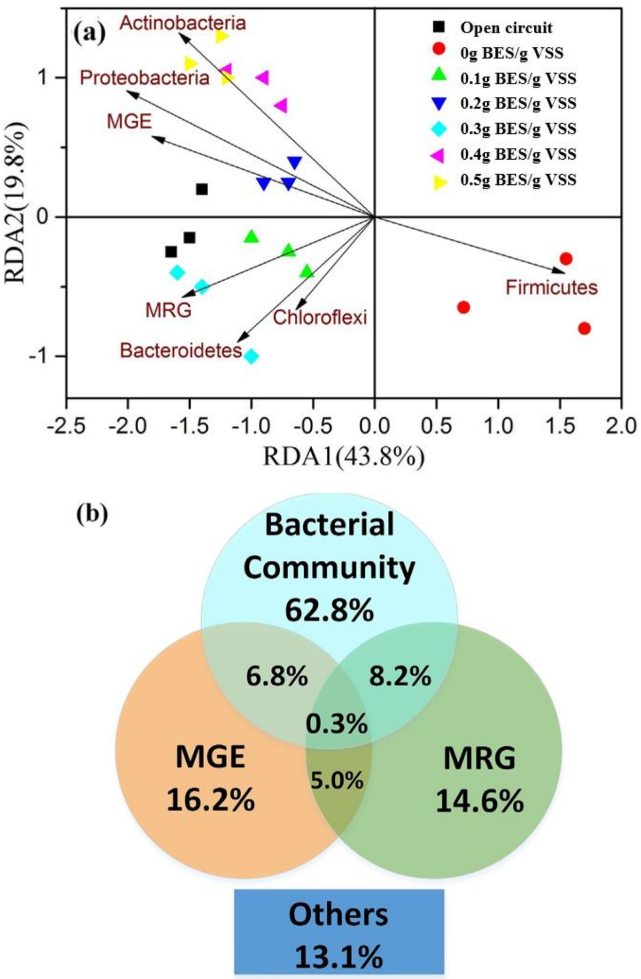

Fig. S5. RDA analysis of all the samples based on ARG subtypes (a) and variation partitioning analysis differentiating the effects of bacterial community, MGEs and MRGs on the ARG abundances (b).

Table S1. The basic chemical characteristic parameters of excess sludge

Character	Value
pH	7.05±0.8
Water content (%)	97.5±0.2
Total chemical oxygen demand (TCOD)	28750±320
Oxidation-reduction potential (ORP)	-82±14
Volatile solid (VS)	9.71±0.88
Dissolved chemical oxygen demand	215.5±4.2
Soluble protein	64.63±2.96
Soluble polysaccharide	18.12±1.85

Table S2. Summary of MGE abundances, including the abundances of plasmids, conjugative transposons, ISs and integrons, in 24 sludge metagenomes

MGEs	Plasmids		Conjugative transposons		Insert sequences		Integrons	
	Reads	Percentage (%)	Reads	Percentage (%)	Reads	Percentage (%)	Reads	Percentage (%)
RS-1	9708	0.330	3971	0.135	12,355	0.420	824	0.028
RS-2	9128	0.290	4501	0.143	13,849	0.440	661	0.021
RS-3	10,182	0.310	4237	0.129	12,153	0.370	887	0.027
0BES-1	7971	0.250	23,500	0.737	9885	0.310	574	0.018
0BES-2	7648	0.260	20,415	0.694	9413	0.320	618	0.021
0 BES -3	6710	0.210	22,943	0.718	9267	0.290	703	0.022
0.1 BES -1	6811	0.210	17,969	0.554	10,055	0.310	584	0.018
0.1 BES -2	7570	0.240	18,579	0.589	6624	0.210	599	0.019
0.1 BES -3	8415	0.260	16,571	0.512	8415	0.260	680	0.021
0.2 BES -1	7302	0.230	15,811	0.498	7937	0.250	476	0.015
0.2 BES -2	6437	0.210	15,356	0.501	6130	0.200	429	0.014
0.2 BES -3	7144	0.230	16,277	0.524	6834	0.220	559	0.018
0.3 BES -1	5498	0.190	14,006	0.484	6656	0.230	492	0.017
0.3 BES -2	6250	0.210	13,154	0.442	6250	0.210	476	0.016
0.3 BES -3	5944	0.220	10,969	0.406	5403	0.200	486	0.018
0.4 BES -1	6363	0.200	12,918	0.406	6045	0.190	541	0.017
0.4 BES -2	4873	0.190	10,258	0.400	5129	0.200	385	0.015
0.4 BES -3	5314	0.210	11,108	0.439	5061	0.200	329	0.013
0.5 BES -1	3497	0.170	8126	0.395	3291	0.160	288	0.014
0.5 BES -2	4375	0.200	8968	0.410	4594	0.210	262	0.012
0.5 BES -3	4061	0.180	8753	0.388	4061	0.180	293	0.013
OC-1	4786	0.200	12,253	0.512	5744	0.240	455	0.019
OC-2	6025	0.230	13,673	0.522	6025	0.230	471	0.018
OC-3	5314	0.210	13,512	0.534	5314	0.210	506	0.020

Table S3 Information of DNA yield and metagenomics sequencing results

Sample name	DNA yield (μg DNA/mL sample)	Reads (100bp)	Tags (~170 bp)
RS-1	8.9894	12,456,134×2	10,567,235
RS-2	8.3246	9,645,100×2	7,762,745
RS-3	10.8465	11,145,780×2	10,563,768
0BES-1	8.5674	12,532,593×2	10,546,321
0BES-2	9.6475	10,346,630×2	9,546,346
0BES-3	8.3451	7,547,769×2	6,523,548
0.1BES-1	6.5783	7,667,634×2	5,673,552
0.1BES-2	7.6786	6,565,645×2	5,789,232
0.1BES-3	9.6487	7,665,453×2	5,652,563
0.2BES-1	7.6785	8,967,897×2	6,453,675
0.2BES-2	6.5467	9,675,642×2	7,623,454
0.2BES-3	7.6524	10,245,673×2	8,657,542
0.3BES-1	5.7867	7,573,465×2	5,674,634
0.3BES-2	7.6765	6,466,853×2	5,635,784
0.3BES-3	8.7623	7,534,346×2	6,546,231
0.4BES-1	5.6897	6,754,575×2	4,652,667
0.4BES-2	6.6546	7,432,676×2	5,675,234
0.4BES-3	7.8756	9,341,643×2	7,634,564
0.5BES-1	5.6785	6,873,986×2	5,723,645
0.5BES-2	6.7632	6,474,865×2	4,652,653
0.5BES-3	5.4673	9,532,673×2	7,845,867
OC-1	7.2568	5,343,897×2	5,734,554
OC-2	6.6747	9,545,764×2	7,236,131
OC-3	6.4568	6,557,834×2	5,723,543

Table S4 Profiles of the microbial community composition in each sample

Phylum	Class	Genus	RS	0BES	0.1BES	0.2BES	0.3BES	0.4BES	0.5BES	OC
Proteobacteria	γ-proteobacteria	*Dokdonella*	3.15	4.63	4.77	4.52	4.50	5.78	4.31	5.35
		Thermomonas	0.37	1.77	2.23	2.88	3.04	1.78	1.51	1.18
		unclassified_Xanthomonadaceae	0.72	1.55	0.94	0.86	0.66	2.34	2.03	3.00
		unclassified_Chromatiaceae	0.14	0.68	0.76	0.82	0.94	0.80	0.66	0.70
		Pseudomonas	0.43	0.61	0.71	0.95	1.14	0.50	0.49	0.22
		BD1-7_clade	0.71	0.49	0.68	0.72	0.85	0.65	0.62	0.45
		unclassified_Xanthomonadales	0.69	0.51	0.68	0.73	0.83	0.52	0.54	0.34
		Nitrosococcus	0.69	0.55	0.67	0.72	0.81	0.65	0.45	0.45
		Escherichia-Shigella	0.52	0.42	0.50	0.59	0.71	0.42	0.43	0.22
		Candidatus_Competibacter	0.40	0.29	0.44	0.50	0.58	0.29	0.20	0.13
		Aeromonas	0.03	0.34	0.53	0.61	0.71	0.30	0.25	0.15
		Dyella	0.01	0.32	0.38	0.46	0.50	0.27	0.38	0.12
		Haliea	0.19	0.29	0.36	0.44	0.51	0.28	0.32	0.13
		Rhizobacter	0.21	0.32	0.35	0.41	0.47	0.28	0.23	0.17
		Aquicella	0.17	0.24	0.30	0.36	0.41	0.27	0.28	0.13
		Acidiferrobacter	0.17	0.24	0.30	0.33	0.38	0.24	0.23	0.13
		Serratia	0.71	0.18	0.25	0.28	0.30	0.17	0.17	0.12
		Acinetobacter	2.44	0.18	0.24	0.26	0.31	0.18	0.16	0.16
		Luteimonas	0.01	0.20	0.23	0.26	0.28	0.21	0.13	0.14
		Thiothrix	0.26	0.20	0.23	0.24	0.30	0.21	0.11	0.14
		Halomonas	0.12	0.09	0.12	0.13	0.15	0.12	0.18	0.10
		Legionella	0.02	0.11	0.11	0.13	0.13	0.13	0.10	0.10

Phylum	Class	Genus	RS	0BES	0.1BES	0.2BES	0.3BES	0.4BES	0.5BES	OC
Proteobacteria	γ-proteobacteria	*Lysobacter*	0.04	0.12	0.10	0.11	0.12	0.14	0.17	0.10
		Cycloclasticus	0.02	0.11	0.10	0.10	0.12	0.13	0.15	0.10
	β-proteobacteria	*Thiomonas*	0.01	1.47	1.94	2.37	2.50	1.55	1.17	1.18
		Comamonas	0.34	1.88	2.29	2.39	2.62	2.05	1.52	1.52
		Azospira	2.43	1.77	2.07	2.35	2.60	1.43	1.17	1.18
		Acidovorax	0.97	1.07	1.32	1.73	1.92	0.96	0.79	0.80
		unclassified_Rhodocyclaceae	1.53	0.57	1.19	1.53	1.70	0.43	0.34	0.24
		Desulfobulbus	1.28	0.86	1.15	1.32	1.48	0.91	0.74	0.56
		Denitratisoma	0.57	0.36	0.55	0.62	0.70	0.28	0.24	0.14
		Ottowia	0.92	0.55	0.63	0.69	0.78	0.59	0.48	0.48
		Dechloromonas	1.16	0.50	0.54	0.60	0.66	0.59	0.40	0.50
		Azoarcus	0.45	0.45	0.51	0.52	0.58	0.49	0.54	0.38
		Achromobacter	0.36	0.41	0.48	0.50	0.55	0.47	0.33	0.34
		Thauera	0.99	0.30	0.40	0.42	0.45	0.29	0.25	0.13
		Ferribacterium	0.83	0.34	0.31	0.39	0.38	0.37	0.37	0.38
		Simplicispira	0.36	0.24	0.29	0.30	0.27	0.24	0.20	0.27
		Sulfuritalea	0.41	0.22	0.28	0.31	0.28	0.21	0.24	0.13
		unclassified_Sphingomonadaceae	0	0.24	0.28	0.29	0.28	0.29	0.19	0.28
		Nitrosomonas	1.17	0.20	0.25	0.23	0.27	0.22	0.18	0.17
		unclassified_Nitrosomonadaceae	2.29	0.19	0.23	0.20	0.20	0.21	0.24	0.15
		Candidatus_Accumulibacter	0.34	0.18	0.21	0.19	0.18	0.23	0.22	0.20
		Polaromonas	0.10	0.18	0.18	0.21	0.21	0.22	0.17	0.22
		Massilia	0.07	0.11	0.12	0.13	0.14	0.13	0.15	0.10

Phylum	Class	Genus	RS	0BES	0.1BES	0.2BES	0.3BES	0.4BES	0.5BES	OC
Proteobacteria	β-proteobacteria	*Variovorax*	0.33	0.10	0.12	0.12	0.10	0.12	0.14	0.10
		Gallionella	0	0.09	0.11	0.11	0.10	0.12	0.13	0.12
		Lautropia	0.16	0.10	0.10	0.11	0.10	0.13	0.09	0.10
		Methylophilus	0	0.10	0.10	0.11	0.10	0.12	0.11	0.12
	α-proteobacteria	*Zymomonas*	0.65	0.66	0.68	0.52	0.47	0.82	0.85	0.79
		Hyphomicrobium	0.37	0.70	0.68	0.57	0.46	1.21	1.02	0.87
		Woodsholea	0.69	0.71	0.66	0.61	0.56	1.13	1.03	0.99
		Novosphingobium	0.46	0.81	0.55	0.44	0.38	1.10	1.07	0.98
		Sphingopyxis	0.27	0.60	0.54	0.45	0.38	1.01	0.93	1.00
		Sphingomonas	0.05	0.46	0.47	0.38	0.31	0.60	0.64	0.64
		unclassified_Caulobacteraceae	0.37	0.44	0.34	0.25	0.23	0.67	0.56	0.70
		unclassified_Rhodospirillaceae	0.38	0.49	0.30	0.27	0.27	0.68	0.56	0.71
		Blastomonas	0.22	0.39	0.28	0.24	0.22	0.55	0.51	0.68
		Bradyrhizobium	0.16	0.44	0.24	0.21	0.19	0.61	0.58	0.61
		Defluviicoccus	0.30	0.42	0.22	0.18	0.15	0.60	0.50	0.61
		Reyranella	0.15	0.24	0.20	0.15	0.12	0.43	0.36	0.41
		Rhodobacter	0.01	0.20	0.18	0.15	0.13	0.25	0.25	0.26
		Defluviimonas	0.11	0.17	0.18	0.15	0.13	0.22	0.18	0.22
		unclassified_Methylocystaceae	0.02	0.17	0.17	0.15	0.13	0.22	0.23	0.22
		unclassified_Holosporaceae	0.10	0.16	0.16	0.15	0.13	0.21	0.18	0.22
		Rhodovulum	0.08	0.17	0.15	0.12	0.10	0.27	0.28	0.26
		unclassified_Methylobacteriaceae	0.12	0.17	0.15	0.13	0.12	0.27	0.19	0.27
		Hirschia	0.24	0.14	0.14	0.12	0.11	0.20	0.15	0.19

Phylum	Class	Genus	RS	0BES	0.1BES	0.2BES	0.3BES	0.4BES	0.5BES	OC
Proteobacteria	α-proteobacteria	*Filomicrobium*	0.24	0.12	0.12	0.11	0.10	0.16	0.11	0.17
		Pedomicrobium	0.05	0.12	0.11	0.10	0.11	0.17	0.14	0.17
		Nordella	0.05	0.11	0.10	0.10	0.10	0.15	0.09	0.15
		Mesorhizobium	0.04	0.12	0.10	0.10	0.10	0.16	0.17	0.19
		unclassified_Hyphomonadaceae	0.12	0.11	0.10	0.10	0.10	0.14	0.17	0.15
	δ-proteobacteria	*Geobacter*	0.21	2.32	2.98	3.73	4.24	2.16	1.86	0.27
		Smithella	0.83	0.57	0.68	0.70	0.87	0.37	0.45	0.13
		Haliangium	1.19	0.25	0.37	0.44	0.57	0.22	0.24	0.13
		Bdellovibrio	0.33	0.25	0.29	0.33	0.37	0.25	0.25	0.19
		Desulfocapsa	0.13	0.86	1.22	1.34	1.32	1.02	0.88	0.14
		Sorangium	0.58	0.25	0.22	0.17	0.12	0.55	0.41	0.92
		Desulfovibrio	0.19	0.88	1.19	1.43	1.59	1.03	0.99	0.47
	ε-proteobacteria	*Sulfurovum*	0.51	0.88	0.72	0.61	0.51	1.27	0.71	1.26
		Sulfurimonas	0.02	0.16	0.10	0.10	0.10	0.28	0.37	0.33
Bacteroidetes	Sphingobacteria	*unclassified_Saprospiraceae*	7.26	9.35	7.07	5.89	5.05	4.91	7.89	7.33
		Crenothrix	0.36	0.92	0.57	0.43	0.38	0.30	0.79	0.86
		Solitalea	0.31	0.79	0.46	0.30	0.27	0.27	0.68	0.75
		Terrimonas	0.11	0.54	0.24	0.13	0.12	0.12	0.41	0.34
		unclassified_Cytophagaceae	0.13	0.22	0.16	0.10	0.10	0.12	0.21	0.19
		Hymenobacter	0.09	0.11	0.11	0.10	0.10	0.12	0.13	0.19
	Flavobacteria	*Flavobacterium*	0.02	0.53	0.41	0.24	0.21	0.22	0.49	0.62
		Chryseobacterium	0.03	0.44	0.29	0.16	0.15	0.15	0.33	0.44
		Segetibacter	0.07	0.27	0.13	0.10	0.12	0.13	0.16	0.13

Phylum	Class	Genus	RS	0BES	0.1BES	0.2BES	0.3BES	0.4BES	0.5BES	OC
Bacteroidetes	Flavobacteria	*unclassified_Chitinophagaceae*	2.35	2.31	2.05	1.93	1.81	1.86	2.20	2.13
		Ferruginibacter	0.81	0.92	0.70	0.67	0.63	0.67	0.87	0.80
		Filimonas	0.16	0.71	0.36	0.11	0.10	0.12	0.45	0.34
	Cryomorphaceae	*Owenweeksia*	1.48	0.20	0.16	0.13	0.17	0.22	0.19	0.36
	Bacteroidetes	*unclassified_Porphyromonadaceae*	0.53	0.33	0.26	0.31	0.38	0.47	0.32	0.38
		Paludibacter	0.14	0.71	0.47	0.11	0.10	0.12	0.60	0.80
Chloroflexi	Anaerolineae	*unclassified_Anaerolineaceae*	12.4	7.26	7.12	6.67	6.13	5.04	7.67	6.27
		Longilinea	0.83	1.05	0.96	0.94	0.81	0.87	1.05	1.33
		Ornatilinea	1.01	0.20	0.28	0.17	0.25	0.20	0.20	0.19
		Leptolinea	0.48	0.95	0.77	0.57	0.46	0.49	1.01	1.39
		Anaerolinea	0.69	0.90	0.73	0.54	0.42	0.37	0.85	0.96
	Caldilineae	*unclassified_Caldilineaceae*	0.15	1.05	0.75	0.37	0.28	0.28	1.09	1.31
Firmicutes	Clostridia	*Clostridium_sensu_stricto_1*	0.17	0.11	0.63	0.80	0.94	1.69	0.37	0.68
		Peptostreptococcus	0	1.16	0.42	0.13	0.12	0.14	0.62	1.03
		Anaerobaculum	0	1.25	1.01	0.81	0.73	0.74	1.17	1.08
		Butyrivibrio	0	1.05	0.74	0.50	0.47	0.51	0.91	0.98
		Thermoanaerobacterium	0	1.77	1.74	1.71	1.32	1.43	1.89	2.03
		Desulfotomaculum	0.07	0.14	0.37	0.48	0.62	0.88	0.27	0.82
		Fusibacter	0.05	0.11	0.14	0.17	0.13	0.14	0.14	0.31
	Bacilli	*Lactobacillus*	0.03	1.85	1.71	1.98	1.56	2.10	1.73	1.61
	Negativicutes	*unclassified_Veillonellaceae*	0.14	1.03	0.44	0.19	0.52	0.21	0.60	0.62
Planctomycetes	Planctomycetacia	*Gemmata*	0.32	0.97	0.86	0.79	0.72	0.82	0.96	1.19
		Pirellula	0.49	1.11	0.94	0.69	0.68	0.71	1.08	1.29

Phylum	Class	Genus	RS	0BES	0.1BES	0.2BES	0.3BES	0.4BES	0.5BES	OC
Planctomycetes	Planctomycetacia	*Planctomyces*	0.84	0.64	0.55	0.53	0.48	0.49	0.66	0.49
		Blastopirellula	0.03	0.12	0.19	0.14	0.16	0.16	0.25	0.49
	Phycisphaera	*Phycisphaera*	0.14	0.15	0.17	0.15	0.14	0.14	0.14	0.20
	Planctomycetia	*unclassified_Planctomycetaceae*	0.62	0.97	1.04	1.04	1.02	1.29	1.17	1.07
Acidobacteria	Acidobacteria	*Blastocatella*	1.36	0.95	0.34	0.37	0.26	0.22	0.97	1.07
		Thermoanaerobaculum	0.28	0.34	0.25	0.24	0.21	0.22	0.30	0.57
		Candidatus_Chloracidobacterium	0.11	0.42	0.23	0.14	0.13	0.14	0.38	0.36
	Solibacteres	*Bryobacter*	0.36	0.43	0.37	0.35	0.30	0.32	0.44	0.86
		Candidatus_Solibacter	0.23	0.43	0.36	0.34	0.30	0.29	0.44	0.68
	Holophagae	*unclassified_Holophagaceae*	0.19	0.20	0.18	0.17	0.13	0.12	0.20	0.30
Nitrospirae	Nitrospira	*Nitrospira*	0.48	1.87	2.95	3.21	3.47	4.31	2.50	2.08
Chlamydiae	Chlamydiae	*unclassified_Simkaniaceae*	0.94	0.82	1.21	1.51	1.80	2.25	0.99	1.18
		unclassified_Chlamydiaceae	0.04	0.15	0.21	0.23	0.33	0.42	0.20	0.51
		Candidatus_Protochlamydia	0.11	0.11	0.19	0.20	0.24	0.32	0.17	0.15
		Candidatus_Metachlamydia	0.09	0.09	0.15	0.17	0.19	0.24	0.14	0.12
		Neochlamydia	0.10	0.09	0.14	0.15	0.16	0.22	0.13	0.09
Verrucomicrobia	Verrucomicrobiae	*Pedosphaera*	0.65	0.29	0.74	0.89	1.04	1.35	0.61	0.67
		Roseibacillus	0.43	0.39	0.61	0.74	0.78	0.98	0.61	0.83
		unclassified_Verrucomicrobiaceae	0.21	0.11	0.18	0.20	0.26	0.31	0.17	0.38
		Prosthecobacter	0.09	0.09	0.16	0.18	0.21	0.27	0.14	0.19
	Spartobacteria	*Chthoniobacter*	0.10	0.15	0.15	0.14	0.13	0.14	0.15	0.19
Ignavibacteriae	Ignavibacteria	*Ignavibacterium*	0.70	0.43	0.56	0.97	0.83	0.88	0.51	0.61
Actinobacteria	Actinobacteria	*Arthrobacter*	0.10	0.23	0.19	0.15	0.14	0.14	0.24	0.14

Phylum	Class	Genus	RS	0BES	0.1BES	0.2BES	0.3BES	0.4BES	0.5BES	OC
Actinobacteria	Actinobacteria	*Iamia*	0.04	0.17	0.16	0.14	0.12	0.13	0.18	0.12
		Solirubrobacter	0.11	0.18	0.14	0.13	0.13	0.14	0.16	0.08
		Atopobium	0.01	0.14	0.13	0.12	0.11	0.12	0.15	0.05
		Acidothermus	0.04	0.11	0.10	0.11	0.10	0.12	0.11	0.03
		Tetrasphaera	0.11	0.09	0.11	0.10	0.12	0.13	0.13	0.14
	Acidimicrobiia	*Candidatus_Microthrix*	0.15	0.24	0.24	0.22	0.22	0.24	0.26	0.18
	Chlorobia	*Chlorobium*	0.06	0.18	0.16	0.13	0.12	0.13	0.18	0.02
Chlorobi	Gemmatimonadetes	*unclassified_Gemmatimonadaceae*	0.88	0.47	0.40	0.35	0.33	0.25	0.47	0.33
Gemmatimonadetes	Armatimonadia	*Armatimonas*	0.10	0.20	0.47	0.30	0.24	0.25	0.19	0.19
Caldiserica	Synergistia	*unclassified_Synergistaceae*	0.09	0.20	0.18	0.16	0.14	0.15	0.20	0.17
Candidatus Saccharimonas			0.74	0.15	0.13	0.12	0.11	0.13	0.16	0.12
Unclassified			2.43	1.98	2.29	2.27	1.66	2.82	2.98	2.77
Others			17.83	4.31	5.01	5.58	5.27	5.35	4.45	4.69

Table S5 Potential ARG hosts revealed by co-occurrence between ARG subtypes and microbial taxa

Genus	ARG type	ARG subtype	Reference
Thiomonas	*sul1*	Sulfonamide	Not available
	sul2	Sulfonamide	Not available
	Hydrophobe_amphiphile efflux-1 (HAE1) family protein	Multidrug	Not available
	mexB	MLS	Not available
	undecaprenol kinase	Bacitracin	Not available
	undecaprenyl-diphosphatase	Bacitracin	Not available
	aminoglycoside resistance protein	Aminoglycoside	Not available
Lactobacillus	*ermT*	MLS	http://ardb.cbcb.umd.edu/cgi/search.cgi?db=T&and0=O&term=ermT&field=af& [47]
	tetPA	Tetracycline	Not available
	tetPB	Tetracycline	Not available
	erythromycin resistance methylase	MLS	[48]
	streptomycin adenyltransferase	Aminoglycoside	Not available
Peptostreptococcaceae	*tetPB*	Tetracycline	Not available
	streptomycin adenyltransferase	Aminoglycoside	Not available
Butyrivibrio	*tetPB*	Tetracycline	Not available

References

47. Costa, B.F., Zarei-Baygi, A., Md Iskander, S., Smith, A.L., Antibiotic resistance genes fate during food waste management - Comparison between thermal treatment, hyperthermophilic composting, and anaerobic membrane bioreactor. *Bioresource Technology* **2023,** 388, 129771.
48. Kong, L., Qi, Y., Shi, X., Variations in antibiotic resistance genes during long-term operation of an upflow anaerobic sludge blanket reactor. Environ Res **2024,** 241, 115755.
